# Protective capacity of neutralizing and non-neutralizing antibodies against glycoprotein B of cytomegalovirus

**DOI:** 10.1371/journal.ppat.1006601

**Published:** 2017-08-30

**Authors:** Anna Bootz, Astrid Karbach, Johannes Spindler, Barbara Kropff, Nina Reuter, Heinrich Sticht, Thomas H. Winkler, William J. Britt, Michael Mach

**Affiliations:** 1 Virologisches Institut, Klinische und Molekulare Virologie, Friedrich-Alexander Universität Erlangen-Nürnberg, Erlangen, Germany; 2 Institut für Biochemie, Friedrich-Alexander Universität Erlangen-Nürnberg, Erlangen, Germany; 3 Nikolaus-Fiebiger-Zentrum für Molekulare Medizin, Friedrich-Alexander Universität Erlangen-Nürnberg, Erlangen, Germany; 4 Departments of Pediatrics, Microbiology and Neurobiology, Children's Hospital of Alabama, University of Alabama, School of Medicine, Birmingham, Alabama, United States of America; Duke University, UNITED STATES

## Abstract

Human cytomegalovirus (HCMV) is an important, ubiquitous pathogen that causes severe clinical disease in immunocompromised individuals, such as organ transplant recipients and infants infected in utero. Antiviral chemotherapy remains problematic due to toxicity of the available compounds and the emergence of viruses resistant to available antiviral therapies. Antiviral antibodies could represent a valuable alternative strategy to limit the clinical consequences of viral disease in patients. The envelope glycoprotein B (gB) of HCMV is a major antigen for the induction of virus neutralizing antibodies. However, the role of anti-gB antibodies in the course of the infection *in-vivo* remains unknown. We have used a murine CMV (MCMV) model to generate and study a number of anti-gB monoclonal antibodies (mAbs) with differing virus-neutralizing capacities. The mAbs were found to bind to similar antigenic structures on MCMV gB that are represented in HCMV gB. When mAbs were used in immunodeficient RAG^-/-^ hosts to limit an ongoing infection we observed a reduction in viral load both with mAbs having potent neutralizing capacity *in-vitro* as well as mAbs classified as non-neutralizing. In a therapeutic setting, neutralizing mAbs showed a greater capacity to reduce the viral burden compared to non-neutralizing antibodies. Efficacy was correlated with sustained concentration of virus neutralizing mAbs *in-vivo* rather than their *in-vitro* neutralizing capacity. Combinations of neutralizing mAbs further augmented the antiviral effect and were found to be as potent in protection as polyvalent serum from immune animals. Prophylactic administration of mAbs before infection was also protective and both neutralizing and non-neutralizing mAbs were equally effective in preventing lethal infection of immunodeficient mice. In summary, our data argue that therapeutic application of potently neutralizing mAbs against gB represent a strategy to modify the outcome of CMV infection in immunodeficient hosts. When present before infection, both neutralizing and non-neutralizing anti-gB exhibited protective capacity.

## Author summary

Human cytomegalovirus (HCMV) is a major global health concern and a vaccine to prevent HCMV disease is a widely recognized medical need. However, no vaccine has been licensed to date. A major obstacle for the development of a vaccine is a lack of knowledge of the nature and specificities of protective responses that should be induced by the vaccine. HCMV is a complex virus containing numerous antigens within the viral envelope that could be targets for protective antibodies. Glycoprotein B (gB) is an important target for neutralizing antibodies and hence an interesting molecule for intervention strategies such as vaccination or passive immunotherapy. We have used the murine model system of CMV (MCMV) to explore the potential of gB-specific antibodies in immunotherapy or prophylaxis. Our results show that anti-gB antibodies can protect immunodeficient hosts from the lethal course of the infection. When used as therapy for established infection, both neutralizing as well as non-neutralizing antibodies showed significant protection with neutralizing antibodies being superior. Among the neutralizing antibodies, protection correlated with sustained *in-vivo* neutralizing activity rather than with the magnitude of the *in-vitro* neutralizing titer. Interestingly, both neutralizing and non-neutralizing antibodies showed comparable protection when given prophylactically i.e. one day before infection with MCMV. Thus, our data indicate that *in-vitro* neutralizing capacity of CMV-specific antibodies may not be reflective of antibody effector functions that provide protection *in-vivo*.

## Introduction

Human cytomegalovirus (HCMV) is an important and ubiquitous human pathogen that is found throughout all geographic locations and socioeconomic groups. Initial infection with HCMV is followed by life-long persistence characterized by episodes of periodic reactivation. While most infections are subclinical in the immunocompetent host, HCMV can cause severe disease and death in immunocompromised patients and newborns infected in utero. As such HCMV is the most frequent viral cause of congenital infection and affects 0.5–2% of all live births worldwide [1,2]. It is the leading infectious cause of childhood sensorineural hearing loss and an important cause of mental retardation [3]. In addition, HCMV is a major cause of morbidity and mortality in recipients of solid organ or stem cell transplants in both the early and late transplant period and is thought to contribute to graft dysfunction leading to graft loss late after transplantation and to overall decreased long term survival in transplant recipients [4,5].

Prevention of end-organ disease and treatment of clinical disease in transplant patients has been achieved using antiviral chemotherapy, although toxicity associated with these compounds and emergence of viruses resistant to currently available antiviral therapies continue to represent a challenge in the clinical care of these patients [6]. In congenitally infected infants, treatment with antivirals has shown some benefit in the most severely affected infants, but the relative benefit of this treatment and the considerable short term and unknown long term toxicity of these agents has resulted in very restricted recommendations for their use [7].

Therefore, prophylactic vaccination has been long argued to be the preferred approach for prevention of HCMV infection and disease in risk groups. However, a prophylactic vaccine remains elusive [8] not least because the nature of the protective immunity to HCMV is far from understood. In general, induction of virus-neutralizing antibodies has been shown to represent a correlate of protection for most effective antiviral vaccines and HCMV will likely be no exception [9]. Thus, identification of major targets for the neutralizing antibody response and characterization of the mode of protection by these antibodies will represent a major step towards development of an effective anti-HCMV vaccine.

Within the envelope of HCMV two proteins or protein complexes have been identified as being the most important targets for the neutralizing antibody response: glycoprotein (g) B and the gH-containing complexes (gH/gL/gO and gH/gL/UL128/UL130/UL131A) [10]. The gH containing protein complexes have recently received attention as potential vaccines since the UL128/UL130/UL131A components of the gH-pentamer complex induce extremely potent neutralizing antibodies during infection [11]. However, these antibodies have restricted activity in that they inhibit infection *in-vitro* of endothelial cells, some epithelial cells and primary cytotrophoblasts but are completely ineffective in preventing infection of fibroblasts and have significantly reduced neutralizing activity against other cell types including trophoblast progenitor cells [11–13]. While vaccination with pentameric complex has repeatedly demonstrated induction of neutralizing antibodies in preclinical models, the protective efficacy of antibodies directed at the pentameric complex in humans remains to be shown [14,15].

Glycoprotein B represents the virion fusion protein for herpesviruses including HCMV [16]. It is essential for the infection of all types of target cells. As such gB remains as an attractive target for inclusion in a human vaccine and has been a major focus of experimental vaccination strategies. In fact, an efficacy study of adjuvanted recombinant gB vaccine (gB/MF59) in postpartum, HCMV-seronegative women suggested an efficacy of approximately 50% protection from acquisition of HCMV infection [17]. Another phase II study in solid organ transplant recipients using the same vaccine showed 50% efficacy in controlling viremia in high-risk patients [18]. In addition, vaccination studies with gB in rhesus macaques and subsequent RhCMV challenge showed significantly reduced RhCMV DNA in plasma [19]. Mucosal immunization with a replication-deficient adenovirus vector expressing murine cytomegalovirus glycoprotein B induced mucosal and systemic immunity [20]. Finally, a number of studies using the guinea pig model demonstrated that congenital infection and mortality in pups was reduced following gB DNA or recombinant protein subunit vaccination strategies [21–23].

The reason(s) for the limited protection in the human gB-vaccine trials are unknown and could be many fold. One reason, among others, could be the induction of an unfavorable ratio of neutralizing antibodies versus non-neutralizing antibodies, which in some cases may even be competitive binders. Available data suggest that vaccination with the gB/MF59 vaccine induced high titers of binding antibodies but more limited titers of neutralizing antibodies [24,25].

Early studies have shown that passive transfer of immune serum obtained from mice infected with MCMV protected recipient mice from a lethal challenge with homologous viruses [26,27]. Using mAbs whose target proteins were not definitively identified, Farrell and Shellam observed some protection in immunocompetent mice [28]. In addition, Jonjic and co-workers demonstrated that B cells, and thus most probably antibodies, were critically involved in restricting dissemination of reactivated virus thus limiting recurrent infection [29]. Our own previous studies have provided evidence that antibodies can provide protection from MCMV-induced pathology in the brain of infected newborn mice [30]. In addition, we could show that in immunodeficient RAG^-/-^ mice, adoptive transfer of memory B-cells or immune serum reduced viral load in organs even when administered 3 days after infection thus exhibiting therapeutic potential [31]. In these studies with polyclonal sera from immune donors, the specificity of protective antibodies and mechanism of protection were not defined. We have initiated studies to define the role of various anti-gB antibodies for protection in the murine CMV model. As in the case of HCMV, gB represents a dominant antibody target during MCMV infection. A panel of monoclonal antibodies against gB was generated, characterized *in-vitro* and their antiviral capacity *in-vivo* was investigated. Our data indicated that therapeutic application of neutralizing anti-gB antibodies has a greater potential to limit virus dissemination than non-neutralizing antibodies. When given prophylactically both neutralizing and non-neutralizing antibodies showed similar protective capacity. *In-vitro*, neutralizing anti-gB antibodies exhibited greater activity in limiting viral cell-to-cell spread which may represent one mechanism for their enhanced protective effect *in-vivo*.

## Results

### Isolation of gB specific monoclonal antibodies

In order to analyze the potential protective capacity of anti-gB antibodies *in-vivo*, we generated a number of gB-specific mAbs. As the primary aim in this experiment was to isolate mAbs with different antiviral capacity *in-vitro*, we used two experimental screening strategies:

1. screening of hybridomas derived from MCMV-infected animals for neutralization and subsequent identification of gB-specificity
2. screening of hybridomas from MCMV-immunized animals for recognition of transiently expressed gB by immunofluorescence assays.

This approach resulted in the isolation of a number of gB-specific mAbs with different antiviral activities *in-vitro* (Table 1). mAbs that were identified by screening with virus neutralization assays had 50% inhibitory concentration (IC50) values of 1–4 μg/ml. These IC50 values were similar to human mAbs directed against HCMV gB [11,32,33]. Most mAbs that were identified from binding assay utilizing transiently expressed gB had IC50s of >20 μg/ml. These mAbs were classified as non-neutralizing.

**Table 1:** Neutralization capacity of glycoprotein B-specific mAbs.

Antibody	IgG subclass	IC50*	IC80*	Source	Screen
18A5	IgG2a	>20	n.d	Balb/c	binding
20H7	IgG3	>20	n.d	Balb/c	binding
5F12	IgG2a	>20	n.d	Balb/c	binding
10H10	IgG1	>20	n.d	Balb/c	binding
97.3	IgG2c	1–2	3–5	C57BL/6	nt
M11	IgG2b	1–2	2–4	C57BL/6	nt
27.7	IgG2c	1–2	4–6	C57BL/6	nt
1F11	IgG2c	2–4	4–6	C57BL/6	binding

* Neutralization was assayed on MEF and concentrations of 50% and 80% neutralization of input virus are given in μg/ml. Values represent mean of several independent tests of independently obtained mAb preparations. n.d.: not defined as 80% neutralization was not reached at concentrations up to 50 μg/ml. Screen: hybridoma supernatants were screened by binding to gB or neutralization (nt). MCMV157luc was used as the reporter virus in all assays in all animals. IC50 for the neutralizing mAbs was similar when MCMVlucMCK2 was used as reporter virus.

**Fig 1:**
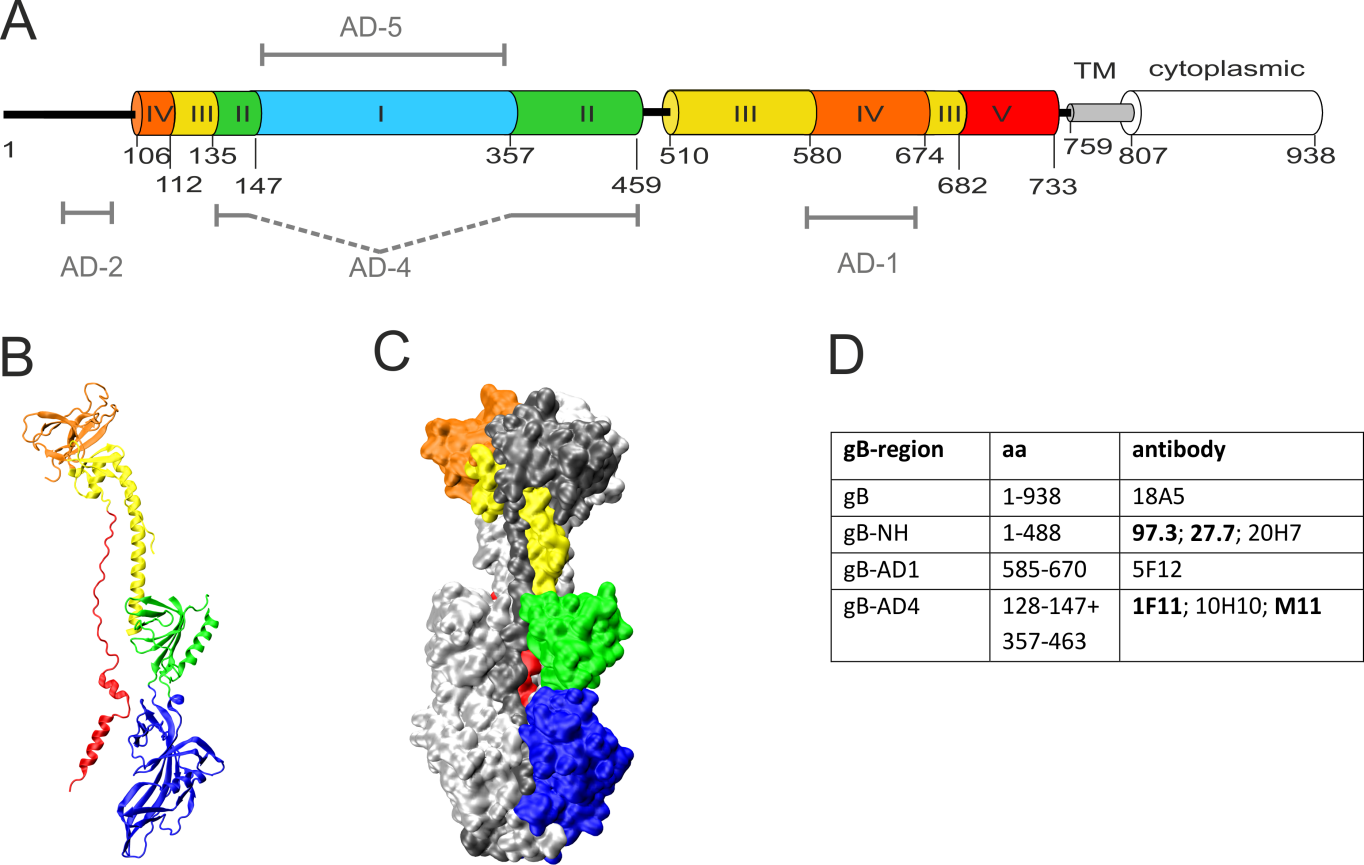
MCMV gB and monoclonal antibodies against MCMV gB. (A) Linear representation of MCMV gB and its structural domains. The regions representing individual structural domains are displayed in different colors in analogy to the HCMV gB crystal structure [36]. TM: transmembrane region. Numbers indicate the beginning of the domains. The antigenic regions (AD) corresponding to identified domains on HCMV gB are indicated. (B) 3D ribbon model of the domain architecture of monomeric gB colored according to (A). (C) Accessible surface representation of a trimer of the MCMV gB model with two protomers coloured in light and dark grey, respectively, and one monomer colored as in A. (D) List of anti-gB mAbs and their binding region on gB. Neutralizing antibodies are shown in bold.

To obtain more information on the binding region within gB, the mAbs were characterized with respect to binding to different antigenic regions on MCMV gB. As a blueprint for generating gB fragments that potentially could harbor antibody binding sites, we followed a strategy that was used to identify conformational epitopes on HCMV gB [34,35]. First, a 3D model of MCMV gB was generated based on the homology to HCMV gB and the crystal structure of HCMV gB [36](Fig 1A–1C). On the amino acid level HCMV gB and MCMV gB show >50% homology thus allowing for the generation of a 3D model of MCMV gB. Structural domains (Dom) that are conserved between the gBs of human herpesviruses were also identified in MCMV gB i.e. DomI-V (Fig 1A–1C). The model also revealed overall structural similarity of the antigenic domains (AD-1, AD-4, AD-5) which were previously identified in HCMV gB (Fig 1A–1C) [32,34].

To determine the mAb binding structures, the protein regions corresponding to AD-1, AD-2, AD-4 and AD-5 of HCMV as well as larger fragments of gB were generated by transient expression in mammalian cells and tested for mAb binding in indirect immunofluorescence analysis [32,34,35]. While no mAbs reacting with AD-2 or AD-5 were isolated, mAb 5F12 bound to AD-1 and mAbs 1F11, 10H10 and M11 bound to AD-4 (Fig 1D). In addition, binding of mAb 97.3 required expression of the NH-terminal part (residues 1–488) of gB while 18A5 required expression of the complete gB protein for recognition. Thus, our panel of mAbs consisted of antibodies with different neutralizing capacity binding to different regions of MCMV gB.

### Therapeutic potential of gB-specific mAbs *in-vivo*

**Fig 2:**
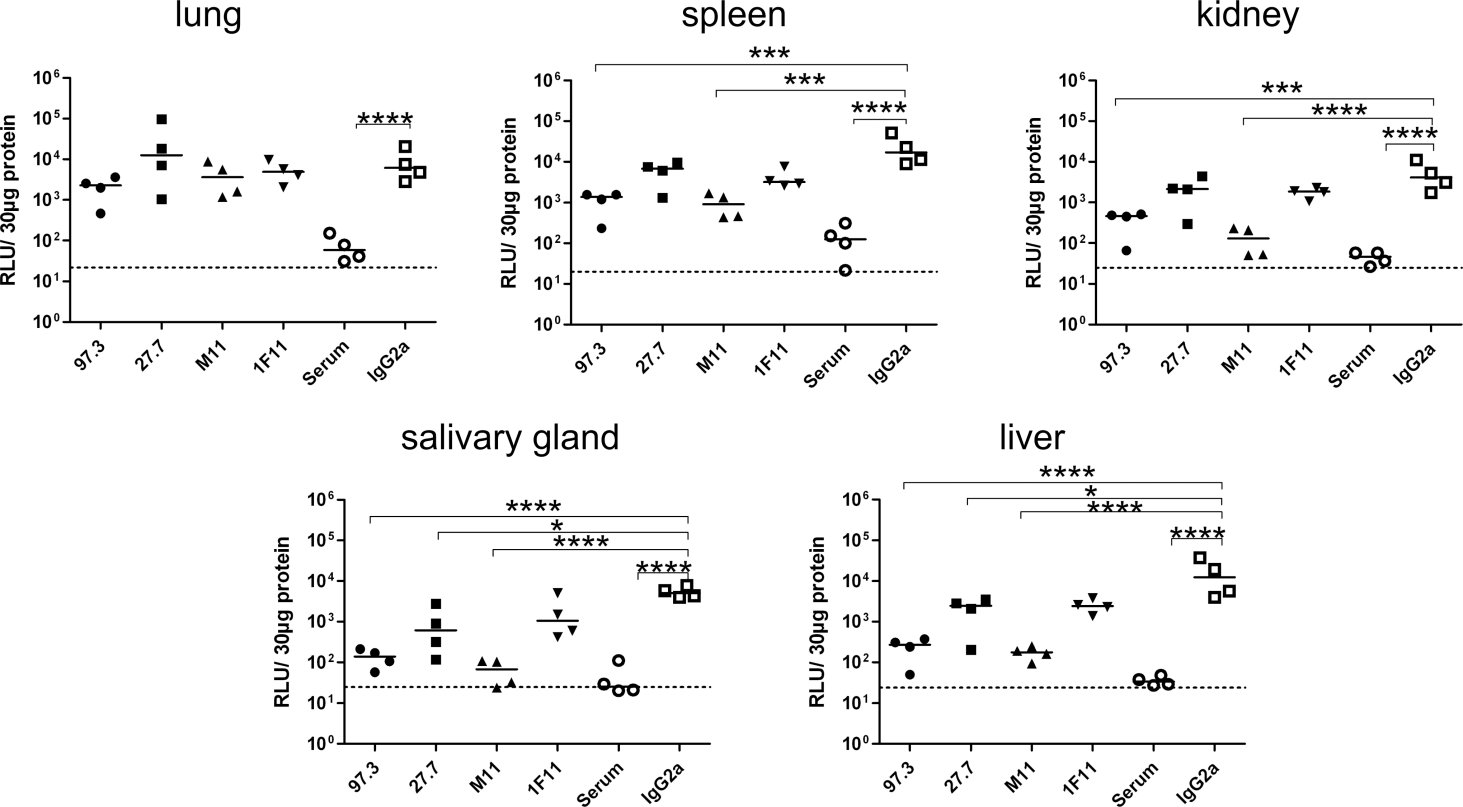
Viral load of RAG^-/-^ mice after therapy with neutralizing antibodies. Mice were infected with 10^5^ pfu MCMV157luc and treated with the indicated mAbs three days after infection. A total of 250 μg IgG or 200 μl serum per animal was injected. Viral load in aliquots of organ homogenates containing 30 μg protein was determined ten days after infection by a luciferase based assay. RLU: relative light units. Statistics: One way ANOVA using Bonferroni´s multiple comparison test *: p<0.05, **: p<0.01, ***: p<0.001, ****: p<0.0001. Dotted line: detection limit. Representative data from 3 independent experiments. Apart from the statistics shown in the figure there were also statistical differences between M11/97.3 vs 27.7/1F11 in liver (p<0.05), kidney (p<0.01) and salivary gland (p<0.05). Differences in lung were not statistically significant.

In a first series of *in-vivo* experiments, we tested mAbs individually for protection from MCMV infection in a therapeutic setting. In these experiments, we used a protocol that was similar to our previous studies which demonstrated protection of immunodeficient mice from the lethal course of MCMV infection by adoptive transfer of polyclonal sera from MCMV-immune donor animals [31]. RAG^-/-^ mice, which do not harbor functional B and T cells, were infected with 10^5^ pfu of MCMV157luc and three days later were treated with mAb or immune serum. On day 10 post infection (p.i.) mice were sacrificed and viral load in organs was quantified. Compared to controls, the viral load was reduced in all organs of animals treated with a neutralizing mAb and this effect reached statistical significance for all organs assayed with the exception of the lung (Fig 2). However, the reduction in viral load that was achieved with mAbs was less pronounced when compared to the effect observed when infected mice were treated with polyclonal serum from MCMV-immune donor animals (supplemental S1 Fig). Interestingly, mAb treatment was able to significantly reduce viral load in salivary glands, the major organ linked to horizontal viral transmission in rodents.

**Fig 3:**
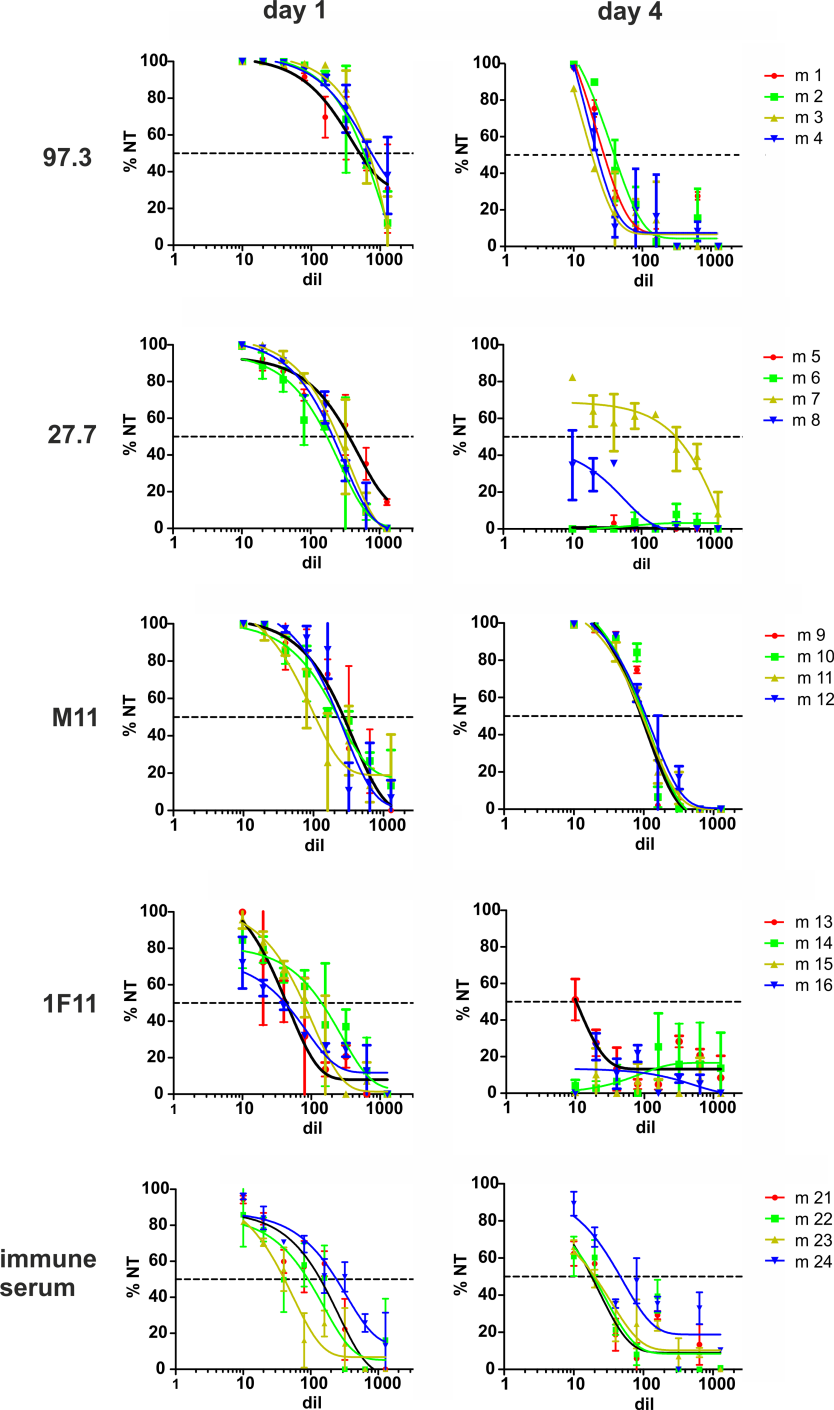
Neutralization titer of sera after adoptive transfer of mAbs. Serum was obtained from infected mice one and four days after injection of mAbs or immune serum. Neutralization titer was determined *in-vitro* on murine embryonic fibroblasts using MCMV157luc. Individual mice receiving the respective mAb are indicated by number and color. Dotted line: 50% neutralization.

Treatment with mAbs 97.3 or M11 resulted in greater reduction in viral load than mAbs 27.7 and 1F11 despite comparable neutralizing activity when assayed *in-vitro* (Table 1) (Fig 2). A potential explanation for the different *in-vivo* activity of 97.3 and M11 versus 27.7 and 1F11 was found when sera from the mAb-treated animals were analyzed for *in-vitro* neutralization one and four days after *in-vivo* administration. One day after mAb injection, sera from most animals resulted in 100% neutralization at a dilution of 1:10 with the exception of animals treated with antibody 1F11 where complete neutralization was reached in serum from only two animals (Fig 3). On day four after antibody transfer, 100% *in-vitro* neutralization was still achieved with sera from animals treated with mAb 97.3 or M11 (Fig 3). In contrast, sera from animals treated with mAbs 27.7 or 1F11 showed lower *in-vitro* neutralization titers which exceeded 50% only in a single animal. The remaining sera had neutralization titers of well below 50% at a dilution of 1:10 (Fig 3). Thus, the most likely explanation for the differences in *in-vivo* protection between 97.3 and M11 compared to 27.7.and 1F11 was reduced antibody concentration in the serum of infected animals treated with mAbs 27.7 and 1F11. Consistent with this explanation was the finding of a statistically significant negative correlation between the decay in neutralizing activity in serum and the reduction in virus titer in organs (supplemental S2 Fig). *In-vitro* neutralization titers of sera from animals treated with immune serum also declined between days 1–4 but exceeded 50% at a dilution of 1:10 in three of four animals.

**Fig 4:**
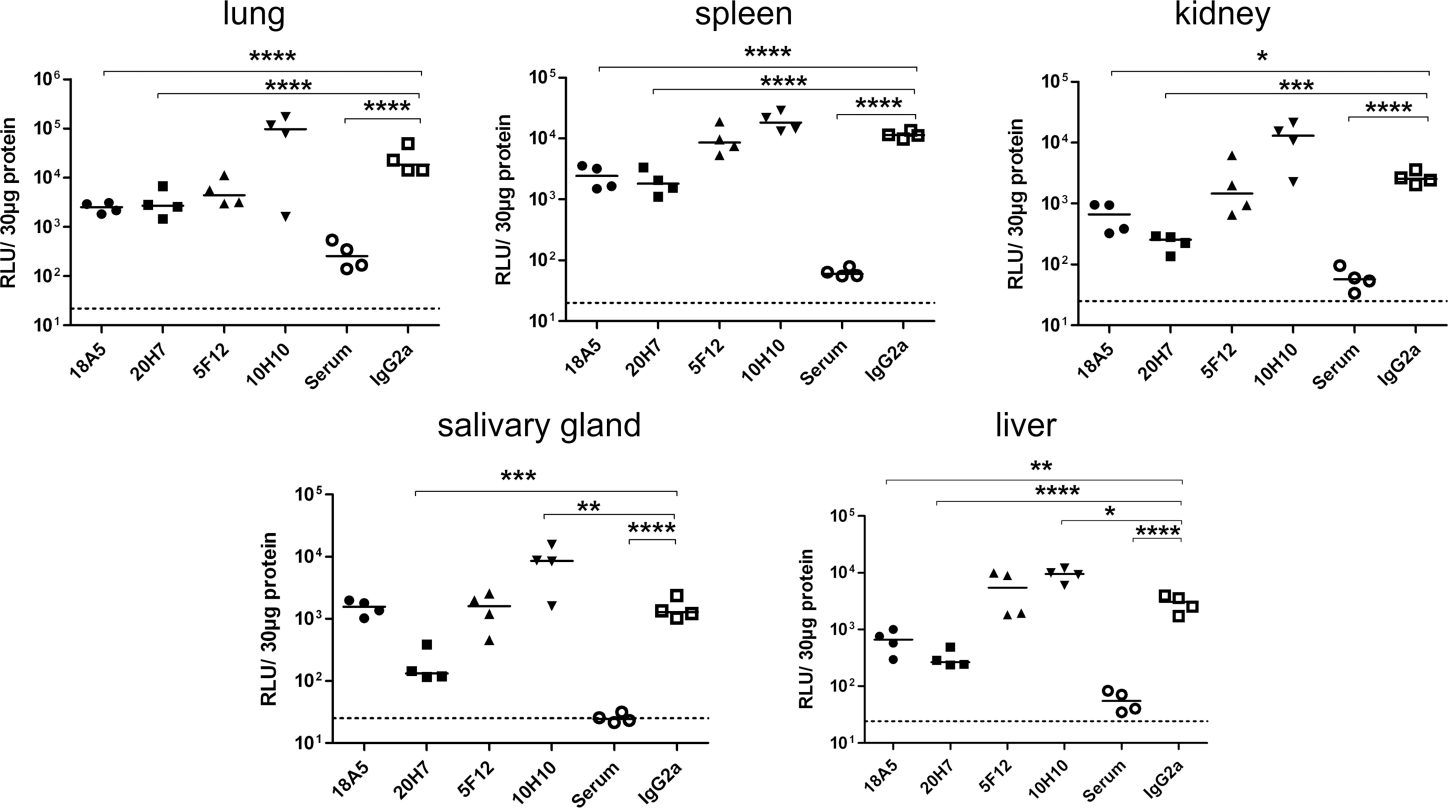
Viral load of RAG^-/-^ mice after therapy with non-neutralizing antibodies. Mice were infected with 10^5^ pfu MCMV157luc and treated with the indicated antibodies three days after infection. A total of 250 μg IgG per animal was injected. Viral load in aliquots of organ homogenates containing 30 μg protein was determined ten days after infection by a luciferase based assay. RLU: relative light units. Statistics: One way ANOVA using Bonferroni´s multiple comparison test *: p<0.05, **: p<0.01, ***:p<0.001, ****:p<0.0001. Dotted line: detection limit. Representative data from 2 independent experiments.

In the next series of experiments, the non-neutralizing mAbs were tested for their antiviral activity *in-vivo*. A reduction in viral load 10 days p.i. was also observed following administration of non-neutralizing anti-gB antibodies (Fig 4). Although the reduction in viral load reached statistical significance, the overall antiviral effect of non-neutralizing mAbs was less pronounced compared to neutralizing mAbs. The extent of reduction in viral load was in the range of 1–10 fold (supplemental S3 Fig) compared to 10–100 fold following administration of the most potent neutralizing mAbs (supplemental S1 Fig). This was particularly apparent for viral load in the salivary glands where only a single non-neutralizing mAb (20H7) was capable of significantly reducing the viral load when compared to the activity of the control immune serum (Fig 4).

**Fig 5:**
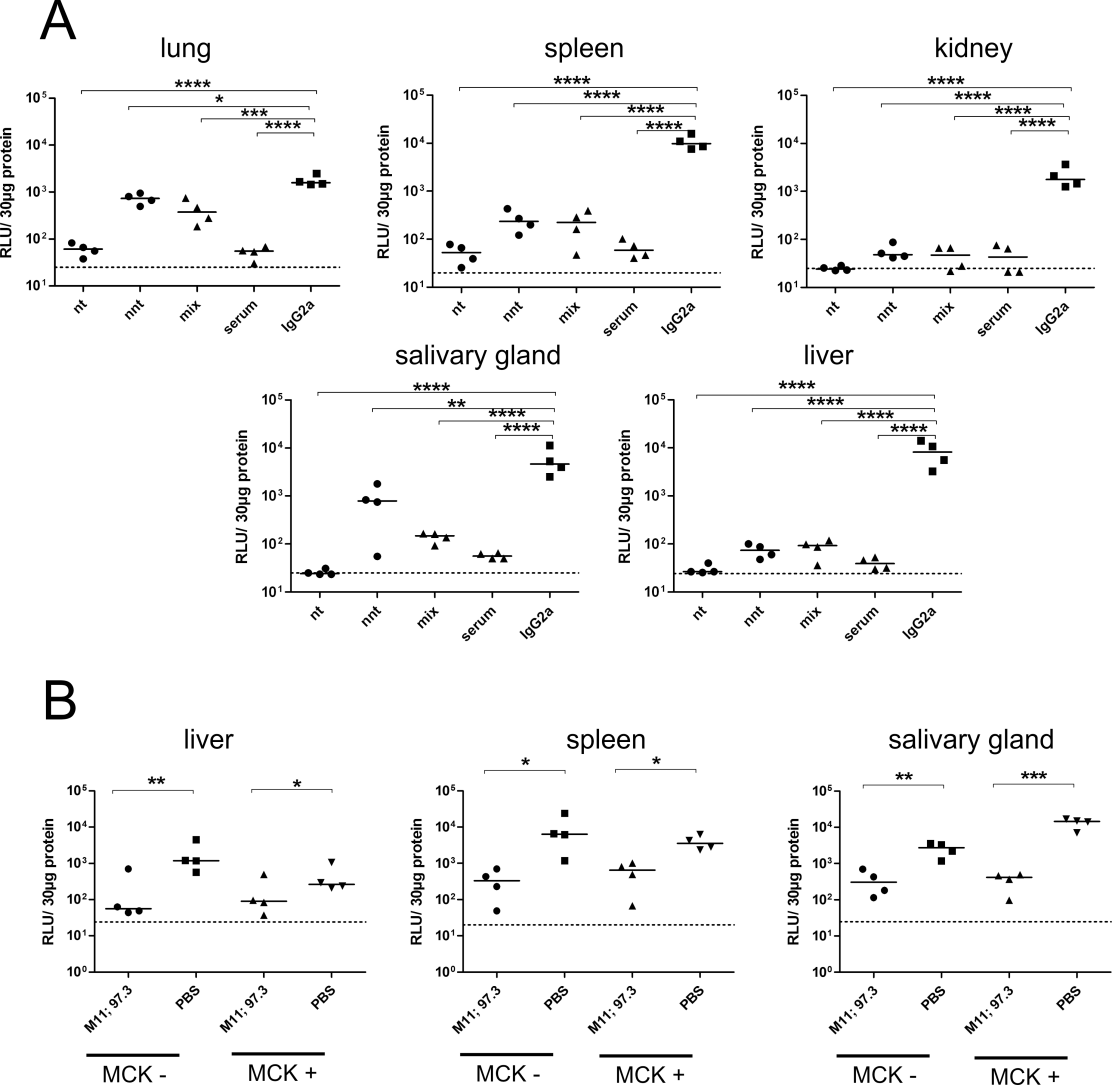
Viral load of RAG^-/-^ mice after therapy with antibody combinations. (A) Mice were infected with 10^5^ pfu MCMV157luc and treated with the indicated combination of mAbs three days after infection. (B) Mice were infected with 10^5^ pfu MCMV-lucMCK2 and treated with the indicated combination of mAbs one day after infection. A total of 250 μg IgG per animal was injected. Viral load in aliquots of organ homogenates containing 30 μg protein was determined ten days after infection by a luciferase based assay. RLU: relative light units. nt: M11+97.3; nnt: 18A5+20H7; mix: M11+97.3+18A5+20H7. MCK-: MCMV containing MCK2 mutation, MCK+: MCMV carrying intact MCK2 gene. Statistics: One way ANOVA using Bonferroni´s multiple comparison test *: p<0.05, **: p<0.01, ***: p<0.001, ****: p<0.0001. Dotted line: detection limit. Representative data from 3 independent experiments.

We also tested if treatment with combinations of mAbs could result in increased *in-vivo* protective activity. To exclude application of mAbs which competed for binding to the same epitope, the mAbs were first tested in competition assays and only mAbs were combined which did not exhibit competitive binding *in-vitro* (supplemental S4 Fig). Animals received either a combination of the two most effective neutralizing mAbs (97.3 and M11) or the two non-neutralizing mAbs (18A5 and 20H7) or a combination of all four mAbs. The total amount of IgG that was injected into the animals was 250 μg/animal in all combinations. As can be seen in Fig 5A, the combination of mAbs 97.3 and M11 was equally effective in reducing the viral load when compared to the activity of polyclonal immune serum. The relative reduction in viral load of mAb combinations was comparable to immune serum and superior to mAb monotherapy (supplemental S5 Fig). In contrast, the combination of non-neutralizing mAbs was less effective in reducing viral burden, although it reached statistical significance when compared to the control IgG2a (Fig 5A). The simultaneous application of neutralizing and non-neutralizing mAbs resulted in intermediate protection as could be expected from the reduction of the amount of neutralizing antibody that was transferred in this four antibody combination.

The MCMV157luc recombinant virus that was used in the above experiments was a derivative of the original BAC construct as described by Messerle et al. [37]. The resulting virus, however, has been shown to carry a mutation in the gene coding for MCK2 which results in altered cell tropism and *in-vivo* dissemination of the virus especially with respect to dissemination to the salivary glands [38]. To explore whether the presence of an intact MCK2 gene would influence the outcome of mAb protection experiments, a new virus was constructed with a repaired MCK2 gene (termed MCMVlucMCK2). This virus was then compared to the MCMV157luc virus in protection experiments. Importantly, the combination of neutralizing mAbs 97.3 plus M11 showed similar protection capacity against the virus carrying an intact MCK2 gene compared to the MCK2-mutated virus. Dissemination of MCMVlucMCK2 to the salivary glands was comparably reduced for both recombinant viruses indicating that the repaired genotype (MCK+) did not influence antibody susceptibility of the virus to neutralizing activity of antibodies during dissemination (Fig 5B).

### Effect of mAbs on virus dissemination *in-vitro*

**Fig 6:**
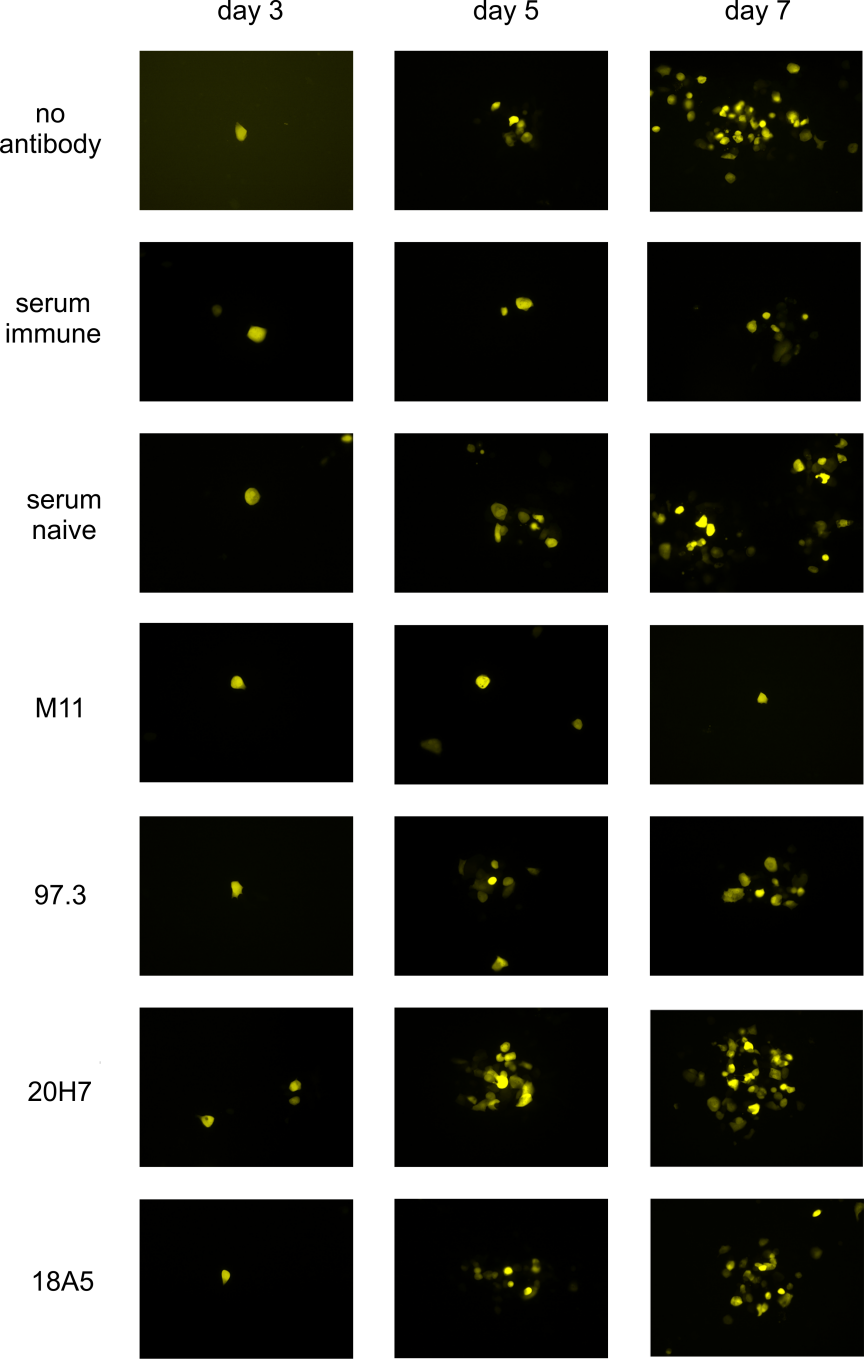
Plaque development in the presence of antibodies. Murine embryonic fibroblasts were infected in 96well plates with 2000 pfu/well of MCMV C3X-gfp. The inoculum was removed 4 h after infection and the indicated antibodies were added. Development of plaques was monitored in live cells on days 3, 5 and 7 after infection. Concentration of mAbs: 20μg/ml, serum dilution 1:100. Numbers of infected cells were counted from 5–7 plaques on day 7 and were found to be (mean and range): No antibody: 25.5 (17–38); immune serum: 4.6 (3–7); non-immune serum: 10.9 (4–18); M11: 1.4 (1–2); 97.3: 7.0 (4–11); 20H7: 14.0 (5–29); 18A5 (10–18). Statistics: (One way ANOVA using Bonferroni´s multiple comparison test) M11 vs no antibody: p<0.001; M11 vs 20H7: p<0.01; M11 vs 18A5: p>0.01; M11 vs serum immune: n.s.; M11 vs serum naïve: n.s.

To define potential mechanism(s) of protection of these antibodies, we performed mAb-mediated plaque inhibition assays *in-vitro*. This assay was selected as surrogate activity for the effect of mAb on viral cell-to-cell spread *in-vivo*. We used a recombinant virus MCMVC3X-gfp, which is a derivative or the original BAC described by Messerle et al. [37] and which expressed the green fluorescent protein (GFP) under control of the HCMV IE enhancer, thus enabling direct visualization of live infected cells [39]. Murine fibroblasts were infected and mAb or serum was added 4 h later and plaque development was monitored between day 3 and day 7 p.i. in live cells. In the absence of antibody only single GFP-expressing cells were observed on day 3 p.i., whereas plaque formation was clearly visible on day 5 p.i. and plaques containing large numbers of fluorescing cells were formed by day 7 p.i. (Fig 6). The addition of non-neutralizing mAbs had little, if any, effect on plaque formation as illustrated by findings from assays using mAbs 20H7 or 18A5 (Fig 6). In the panel of neutralizing mAbs, M11 completely prevented plaque formation while the remaining mAbs clearly reduced the number of infected cells per plaque, including mAb 97.3 (Fig 6). Polyclonal immune serum had an intermediate effect with respect to numbers of infected cells within plaques whereas serum from naïve mice failed to limit plaque formation in this assay.

### Prophylactic capacity of gB-specific mAbs

**Fig 7:**
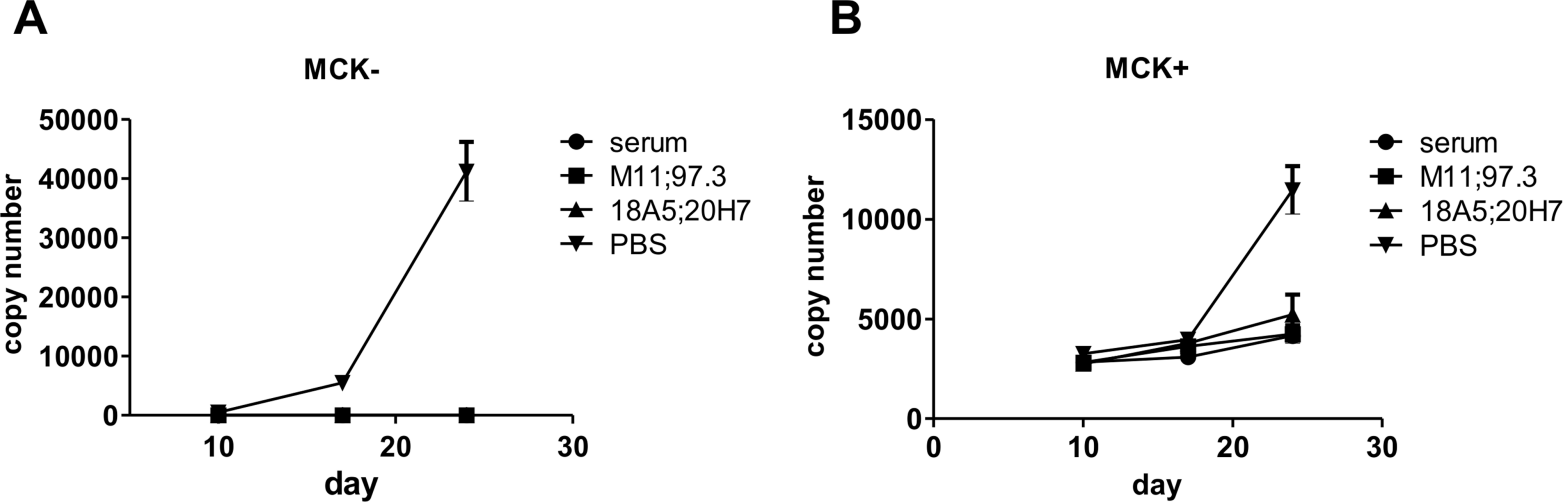
Protective capacity of mAb combinations following prophylactic application. A total of 250 μg IgG (M11+97.3 or 18A5+20H7) per mouse or 200 μl serum/PBS was injected one day before infection with 10^4^ pfu of MCMV157luc (A) or MCMVlucMCK2 (B). Blood was taken at the indicated time points and qPCR performed. n = 4 in antibody treated groups and n = 3 in the PBS treated group. Values represent mean (SEM) of all mice within one group and duplicate determinations per sample. MCMV genome copy number is given per 1μg total DNA. Detection limit: 1 copy/50ng total DNA.

We next tested the protective capacity of the mAb combinations when transferred before infection. RAG^-/-^ mice were given 250 μg total IgG one day before infection with 10^4^ pfu MCMV157luc. On days 10, 17 and 24 p.i. blood was taken and viral DNA was quantified by quantitative real time PCR (qPCR). In PBS treated mice DNA copies increased from about 500 copies at day 10 to >40 000 copies per μg of total DNA on day 24, indicating an ongoing viremia (Fig 7). In immune serum-treated mice, viral DNA was detectable with low (2–10) copy numbers in some animals. Application of the mAb combinations, either neutralizing or non-neutralizing, resulted in the clearance of viral DNA from the blood at any time point p.i. (Fig 7A). We next repeated this experiment utilizing the MCK2+ virus (MCMVlucMCK2) to determine if the presence of the MCK2 viral gene could result in altered viral replication and pathogenesis in infected mice that would in turn alter the antiviral activity of mAbs administered in a prophylactic protocol. Infection with the MCK2+ virus resulted in higher viral DNA copies in the blood compared to the MCK2- virus. The increased copy number of the MCK+ virus in the blood of the infected animals was not unexpected as MCK2 has been shown to increase the number of infected leukocytes and facilitate the recruitment and infection of monocyte/macrophages [40–42]. Importantly, however, the viral load in animals following application of neutralizing or non-neutralizing mAbs was similar to the activity of immune serum, again indicating that both classes of antibodies have similar antiviral activity when given prophylactically. (Fig 7B)

In a second experimental approach, survival was monitored following infection with the original MCMV157luc virus and prophylactic administration of a different set of anti-gB mAbs, namely the therapeutically less potent neutralizing mAb 1F11 or the non-neutralizing mAb 5F12. Mice treated with control IgG2a succumbed to the infection by day 40 p.i. (supplemental S6 Fig). In contrast, about 80% of mice receiving gB-specific mAbs or immune serum survived the infection until day 100. Again, the non-neutralizing mAb 5F12 was as effective as the neutralizing mAb 1F11 in prolonging survival of infected mice when given prophylactically.

Taken together, these data indicate that the presence of antibody before the infection can result in significant protective capacity largely independent of the *in-vitro* neutralizing activity of the antibody.

## Discussion

We have previously shown that serum from MCMV-infected animals can protect B and T cell deficient hosts from lethal MCMV infection, thus demonstrating that passively acquired antibodies present in immune serum are protective *in-vivo* in the absence of *de-novo* adaptive responses to MCMV [31]. The antiviral antibody responses induced during MCMV infection is complex and includes antibody responses against a large number of virion structural and virus-encoded non-structural proteins. Only some of these responses have measurable *in-vitro* and/or *in-vivo* antiviral effector activity with the most well studied antiviral responses being directed against envelope glycoproteins. Following both MCMV and HCMV infections, anti-gB, gH/gL, and gM/gN antibody responses can be demonstrated and antibodies against gB, gH, or gN have been shown to neutralize virus *in-vitro* [10]. In the case of HCMV, antibodies against a pentameric complex consisting of gH/gL/UL128/UL130-131a have been shown to exhibit potent *in-vitro* neutralizing activity in assays utilizing epithelial and endothelial cell targets and a limited number of other cell types, but not in more permissive cell types such as fibroblasts [11–13]. Although we have isolated anti-gH and anti-gN virus neutralizing antibodies from MCMV infected mice, the goal of the current study was to evaluate gB-specific mAbs for their protective potential to model the role of gB antibodies during HCMV infections since gB represents a dominant antigen for the induction of antibodies during HCMV infection. In addition, we studied the *in-vivo* activities of both neutralizing as well as non-neutralizing anti-gB antibodies as both types of antibodies are induced during HCMV infection [32].

Antibodies selected for study included two groups of mAbs that were classified *in-vitro* as neutralizing or non-neutralizing as antibodies exhibiting these *in-vitro* activities were representative as a sample of anti-gB responses in MCMV infected mice. This different antiviral activity is also consistent with data from studies that have described antiviral neutralizing and non-neutralizing anti-gB antibodies against HCMV [32]. In addition, the antigenic regions that were recognized by the MCMV gB-specific mAbs were similar to those recognized by anti-HCMV gB mAbs, providing further support for the relevance of results from studies of the protective activities of anti-gB antibody responses in MCMV infected mice as a model of the role of anti-gB antibodies in HCMV infections [32,43].

In experiments designed to determine the therapeutic potential (treatment) of the anti-gB mAbs, we used a rigorous protocol in which gB mAbs were given three days after an i.p. infection of immunodeficient RAG^-/-^ mice with 10^5^ pfu of MCMV157luc. The quantity of antibody that was transferred per animal roughly translates to 10 mg/kg, a concentration of antibody that has been has been used clinically in humans [44,45]. In RAG^-/-^ mice, in addition to the B and T cell deficiency, virus control by NK cells was largely eliminated secondary to the deletion of the m157 gene that encodes a NK activating ligand [46]. Within the experimental time frame of 10 days following infection, MCMV had disseminated to all organs including the salivary glands, a privileged anatomical site of cytomegalovirus immune evasion and persistence which has relevance for horizontal transmission of CMVs [47]. Monotherapy with neutralizing mAbs showed significant reduction in viral load in all tested organs with exception of the lungs. Interestingly, the viral load in salivary glands was also reduced significantly by passively transferred virus neutralizing antibodies, indicating inhibition of virus dissemination during secondary viremia [48]. The reduction in viral load was correlated quantitatively with the concentration of mAbs that was maintained in the serum of treated animals. Animals that received mAbs that exhibited an accelerated *in-vivo* decline in the serum concentration, such as 27.7 and 1F11, were poorly protected as compared to animals treated with antibodies that maintained sustained mAb concentration. We can only speculate on the reasons for the differences in the serum decay of the different mAbs *in-vivo* since this process is complex, but obvious mechanisms could include off-target binding and/or immune complex formation resulting in increased rates of IgG elimination [49]. Significant differences in *in-vivo* half-life of transfused IgG has also be observed in other systems [50]. Regardless of the mechanism(s) of increased mAb elimination from the serum of MCMV infected mice, our findings indicated that mAb monotherapy that resulted in sustained high titers of circulating MCMV neutralizing antibodies in infected mice resulted in significant protection from lethal MCMV infection in immunodeficient mice. Importantly, this finding established an *in-vivo* property of antiviral antibodies that were required for optimal protection from viral dissemination and argued that simple classification of antibodies as neutralizing and non-neutralizing in *in-vitro* assays may not be fully predictive of *in-vivo* antiviral activity.

Results from studies utilizing non-neutralizing antibodies argued that there was not an absolute requirement for potent *in-vitro* neutralizing activity in order to achieve protection *in-vivo*. Although the reduction in viral load following administration of non-neutralizing mAbs was less than that seen following transfer of neutralizing mAbs, the reduction in viral load was still statistically significant for antibodies such as 18A5 and 20H7 (compare supplemental S1 and S3 Figs). Similar to the results following treatment with neutralizing mAbs, we observed differential activities of individual mAbs with respect to reduction of viral load in mAbs treated animals. Whether the differential protection was also based on variation of *in-vivo* serum concentration of the individual mAbs could not be determined as we have no quantitative assay to specifically quantify the concentration of these mAbs in serum. In any case, non-neutralizing mAbs provided remarkable protection *in-vivo* in the absence of appreciable *in-vitro* neutralizing activity. The finding that non-neutralizing antibodies can provide protection from viral infections has also been observed previously [51] and has recently become a major area of interest in studies of protective antibodies against influenza virus and HIV infections [52,53].

Effector functions associated with protection by our panel of mAbs are likely to be complex and remain undetermined at this time. Interaction of the antibody Fc-fragment with Fc-receptor bearing cells, resulting in antibody-dependent cell mediated cytotoxicity (ADCC) or antibody-dependent cellular phagocytosis (ADCP) may represent important mechanisms (reviewed in [54]). However, affinity of individual Fc-receptor molecules for the different IgG subclasses molecules has been shown to be different. Thus, for our panel of mAbs representing different IgG subclasses the contribution of Fc-receptor interactions could vary. For example, 20H7, being an IgG3, will not be bound by any of the known Fc-receptors [55]. Moreover, CMVs also express virally encoded Fc-receptor molecules that potentially add even more complexity to the contribution of Fc-receptor dependent antiviral antibody activity to the interpretation of our findings (reviewed in [56]).

Finally, in addition to Fc-receptor binding, complement activation *in-vivo* may also be operative for some of the mAbs in our panel. However, complement binding and activation is also different for individual IgG subclasses (reviewed in [57]). Moreover, herpesviruses including CMVs have developed a number of strategies to evade complement mediated functions. Among those are incorporation of complement control proteins in the virion particle or inhibition of complement-mediated lysis [58,59].Thus, additional studies will be required to elucidate the mechanism(s) by which the individual mAbs represented in our panel will provide protection.

A potentially important mechanism of virus dissemination *in-vivo* is by cell-to-cell spread, a mechanism that could be limited by antiviral antibodies. Herpesviruses, including CMVs, are believed to have the capacity to spread to contiguous cells without having to transit via the extracellular space [60]. In the case of CMV infections, *in-vivo* cell-to-cell spread is considered to be a major route of viral dissemination. However, the routes and molecular mechanisms through which CMVs spread from cell-to-cell *in-vivo* remain poorly defined [61,62]. Regardless of the mechanism(s) of cell-to-cell spread, *in-vitro* studies using plaque formation/expansion has been suggested to be a surrogate for cell-to-cell spread. Our data indicate that cell-to-cell spread of MCMV can be inhibited by a subset of antiviral antibodies from a group of mAbs with comparable neutralizing activity when measured *in-vitro* with cell-free virus, suggesting that the requirements of mAb activity for inhibition of cell-to-cell spread could be distinct from those characteristics of mAbs required for neutralization of cell free virus infection. Consistent with additional effector functions of virus neutralizing mAbs was the finding that a combination of M11 and 97.3 was more potent in *in-vivo* protection than either antibody transferred individually. However, synergism in *in-vitro* virus neutralization between these two antibodies could not be demonstrated (supplemental S7 Fig). Also, the serum neutralizing capacity when both antibodies were given in combination as well as the concentration *in-vivo* of neutralizing activity did not differ from that observed in mice treated with an individual antibody (supplemental S7 Fig). However, the finding that they have different inhibitory activity potency when compared in an *in-vitro* plaque formation inhibition assay indicated that their mode of action *in-vivo*, apart from neutralization of free virus, could be different and potentially was linked to the increased protective activity of the combination of mAbs. Finally our findings also suggest that inhibition of plaque formation *in-vitro* may represent a more informative assay for prediction of *in-vivo* protection than neutralizing capacity determined by an *in-vitro* assay using cell free virus.

Perhaps one of the most interesting and unexpected findings in this study was that for the panel of mAbs tested there was no difference in the *in-vivo* antiviral activity between non-neutralizing and neutralizing mAbs when these mAbs were used in a prophylactic protocol. Moreover, monotherapy with single antibodies from these mAb combinations also provided significant protection from the lethal course of the infection in RAG^-/-^ mice. Although these results will require further studies to define mechanisms of protection provided by neutralizing and non-neutralizing antibodies in this model system, there are several possible explanations that could account for these results. Importantly, there is a fundamental difference in antibody-virus interaction between a primary virus inoculation and an established infection when mAbs are used in a therapeutic protocol. In our experiments, the virus was inoculated intraperitoneally and free virus spreads within the first hours via a haematogenous route to the spleen and liver [63]. Thus, disseminating virus comes into contact with circulating antiviral IgG. As both, neutralizing and non-neutralizing mAbs can bind to free virions, antibody effector functions mediated via the Fc could prevent infection of the first cellular target of infection. As the antibody coated virus is transported to structures such as the spleen which are rich in cells carrying Fc-receptors it could be eliminated via Fc-mediated effector functions. Whether an antibody has neutralizing function or not could be less critical in terms of its protective activities during these early events of infection. Virus neutralizing activity could play a more important role in control of virus spread at later time points during infection i.e. during cell-to-cell spread in infected tissues and/or secondary viremia.

Vaccination trials in humans using an adjuvanted gB have provided conflicting evidence of protection from community acquisition of HCMV [17]. In the initial report, three doses of the gB vaccine limited acquisition of HCMV in a group of women and although differences between vaccine recipients and placebo controls were observed, the statistical difference between the two groups was not robust [17]. In a follow-up vaccine trial in adolescent females, there was no statistically significant difference in acquisition of HCMV between gB vaccine and placebo recipients [64]. In contrast, our findings demonstrated that anti-gB antibodies have potent protection capacity *in-vivo*, particularly when used as prophylaxis to limit infection. There are several explanations that could account for this difference:

1. immunization with the adjuvanted gB vaccine induced high titers of gB-binding antibodies but the titers of neutralizing antibodies were not systematically investigated in the reported vaccine trials [25,64].
2. the vaccine antigen in the adjuvanted gB vaccine does not represent the native gB form. It was engineered as a truncated protein to increase solubility and to simplify its production. In addition, the proteolytic cleavage site was mutated to eliminate proteolytic cleavage between the ectodomain and transmembrane domains. These modification resulted in a post-fusion conformation of gB, a form that may be considerably different from the prefusion conformation [16,65], thus potentially inducing a different set of antibodies as compared to anti-gB antibodies generated during infection, including antibodies directed at the surface of virions such as the antibodies used in our study.
3. the vaccine protein was emulsified in an adjuvant which could further modify the protein conformation in contrast to our study which used non-adjuvanted virions.
4. HCMV gB strain variations could have limited the protective capacity of vaccine induced antibodies, although at the amino acid level, gB is a highly conserved protein between HCMV strains [66].

Taken together, our study has provided convincing evidence that a subset of antibodies directed against gB of MCMV raised either following infection or after immunization with intact virions can provide significant protection *in-vivo* when transferred into MCMV infected mice either in a prophylactic or treatment protocol. Potent *in-vitro* neutralizing activity seems not to be an absolute prerequisite for this effect. Whether antibodies with these specificities and activities can be generated following vaccination will be investigated in future studies.

## Materials and methods

### Mice

RAG^-/-^ mice were obtained from in-house breeding based on mice from Charles River and maintained under specific pathogen-free conditions. In experiments involving therapeutic application of mAbs, mice were infected with 1 x 10^5^ plaque forming units (pfu) of MCMV157luc or MCMVlucMCK2 by intraperitoneal (i.p.) infection. In experiments involving prophylactic application of antibodies, 10^4^ pfu MCMV157luc or MCMVlucMCK2 was used. *In-vivo* bioluminescence imaging was done exactly as described [31].

### Ethics statement

All experiments were conducted in accordance with institutional guidelines for animal care and use. The experiments were approved by the Regierung von Mittelfranken (Government of Frankonia) approval 54–2532.1-57/12 and adhered to the EEC Council Directive 2010/63/EU. The animal facility of the University of Erlangen-Nürnberg is approved by the Dept. of Health & Human Services, USA, approval number A5903-01.

### Cells and viruses

Mouse embryonic fibroblasts (MEF) and ST-2 cells were cultured in DMEM medium (Life Technologies, Germany) supplemented with 10% fetal calf serum (FCS) (Sigma-Aldrich, Germany), glutamine (100 mg/ml), and gentamicin (350 mg/ml).

All virus strains were derived from the original MCMV BAC as described by Messerle [37]. MCMV157luc was propagated and purified as described [31]. The MCK2 mutation in MCMV157luc was repaired as reported by Jordan et al. [38] resulting in virus MCMVlucMCK2. Virus titer was determined by end-point titration using indirect immunofluorescence. Briefly, serial dilutions of viral preparations were used to infect MEF that had been seeded in 96-well plates (12 000 cells/well). Two days later, cells were fixed with ethanol and infected cells were stained and quantified using the monoclonal antibody Croma101, which is specific for the viral immediate early protein 1 of MCMV. MCMV-C3X-gfp which expresses the green fluorescent protein (GFP) under control of the HCMV immediate early promoter was a kind gift from M. Messerle, Hannover, Germany [39].

### Monoclonal antibody generation, IgG subclass determination and biotinylation

Immortalized antibody-producing B-cell lines were generated from the spleens of infected or immunized donor mice by conventional hybridoma technology. Briefly, C57BL/6 mice were i.p. infected with 1x10^6^ pfu MCMV157luc and spleen cells were harvested 4–6 weeks after infection. One week before harvest of the spleen, the animals were boosted with 5 μg of UV-inactivated MCMV157luc virions. Following this protocol the Mabs 1F11, 27.7, M11 and 97.3 were obtained from three different fusions. Mabs 18A5, 20H7 were isolated from Balb/c mice that were treated with an identical protocol. Mabs 5F12 and 10H10 were obtained following immunization of a Balb/c mouse (three times 5 μg each of UV-inactivated MCMV157luc virions i.p. and intervals between injections of at least 4 weeks.) Three to four days after the last immunization, spleens were removed and splenic cells (100–200 x10^6^ cells) of the donor mouse were fused with 50–100 x 10^6^ SP2.0 cells. Cells were seeded in 96 F-bottom cell culture microplates in 150 μl medium per well. 8–10 days later, supernatants were tested for neutralization (from mice that were infected) or virion binding antibodies in an ELISA (from mice that were immunized with UV-inactivated virions). Clones of interest were subcloned using a Beckman Coulter MoFlo cell high-speed sorter®. Following additional rounds of subcloning, hybridoma supernatants were characterized by ELISA, indirect immunofluorescence using transiently expressed gB and neutralization. mAbs were purified in-house by protein A chromatography or prepared and purified by BioXCell (USA).

IgG subtypes were determined using a mouse immunoglobulin panel (Southern Biotech, Germany, Cat.No:5300–01) and an IgG2c isotype control antibody (GeneTex, USA, Cat.No:GTX35043) as coating reagents in standard ELISA assays. Standard IgGs were coated at a concentration of 100ng/well in 96 well plates and compared to undiluted samples from hybridoma supernatants. Assays were developed using a Southern Biotech SBA Clonotyping System-HRP (Cat.No.:5300–05) according to the manufacturer´s suggestion complemented by Goat Anti-Mouse IgG2c HRP to detect IgG2c (Southern Biotech Cat.No.:1079–05). Biotinylation of purified mAbs (500 μg each) was carried out using the EZ Link® Sulfo-NHS Biotinylation Kit (Thermo Fisher Scientific, Germany) according to the manufacturer’s instructions. Biotinylation was confirmed in ELISA assays using the mAbs as coating reagent and HRP conjugated Streptavidin as detecting reagent.

### Measurement of organ luciferase-activity

Organs were harvested and snap frozen in liquid nitrogen. For determination of virus titer, organs were thawed and homogenized in Glo Lysis Buffer (Promega, Germany) using a Precellys 24 homogenizer (Peqlab Biotechnologie, Germany). Homogenates were centrifuged at 4°C for 10min at 16000xg and protein concentration was determined in the supernatant using a BCA Protein Assay Kit (Perbio Science, Germany). 30 μl Glo lysis buffer containing 30 μg protein of lysates were transferred into white 96 well LIA-plates (Greiner Bio-one, Germany). 50 μl assay buffer (15 mM KH_2_PO_4_, 25 mM glycylglycine, 1 M MgSO_4_, 0.5 M EGTA, 5 mM ATP, 1 mM DTT) per well was added. Injection of 50 μl D-luciferin- (P.J.K., Germany) solution per well (In 25 mM glycylglycine, 1 M MgSO_4_, 0,5 M EGTA, 2 mM DTT and 0,05 mM D-Luciferin) and detection of chemiluminescence was performed by a Centro LB 960 Luminometer (Berthold Technologies, Germany). MicroWin2000 Software (Mikrotek Laborsysteme, Germany) was used for analysis.

### Measurement of viral DNA copies by quantitative real time PCR

To measure virus copy numbers in peripheral blood, DNA was isolated from 200 μl of EDTA-blood using the QIAamp DNA blood kit according to the manufacturer’s protocol (QIAgen, Germany). For MCMV-specific qPCR, 50 ng of the isolated DNA was subjected to a 20-μl reaction mixture containing 10 μl 2x TaqMan PCR Mastermix (Applied Biosystems, Germany), 10 μM probe and 5 μM of each primer. Primers and probe for the detection of MCMV were based on the MCMV ie1/4 exon 4 sequence (forward primer: 5′-TGCCATACTGCCAGCTGAGA-3′; reverse primer: 5′-GGCTTCATGATCCACCCTGTT-3′; and probe: 5′-CTGGCATCCAGGAAAGGCTTGGTG-3′).

### *In-vitro* neutralization assay

For *in-vitro* neutralization, serial dilutions of sera or monoclonal antibody were incubated with 1200 pfu MCMV157luc for 1h. The mixture was added to ST-2 cells that were seeded at a density of 1.2x10^4^ the day before in 96-well plates. Following incubation for 4h the culture medium was changed and infection continued for 48hrs. Thereafter cells were lysed in 100 μl Glo lysis buffer and 30 μl were used to measure luciferase activity as described above. Sera were not heat inactivated and no exogenous complement was added.

### Transient protein expression and image analysis to identify binding regions on MCMV gB

To express MCMV gB, the coding sequence from orf m55 strain Smith (GenBank accession number NC_004065.1) was inserted into pcDNA3. The plasmids encoding fragments of gB were constructed by inserting the appropriate DNA fragment into the vector pcUL132-sig-HA. This pcDNA3.1 (Invitrogen, Germany) based plasmid contains the coding sequence of the HCMV gpUL132 authentic signal sequence aa 1–27, followed by the coding sequence for the HA-epitope YPYDVPDYA [67].

Cos7 cells (5x10^4^ per well) grown in 24-well plates on 15-mm glass coverslips were transfected with 0.8 μg plasmid DNA using Lipofectamine (Invitrogen, Germany). 48 hours after transfection, cells were fixed and permeabilized with ice cold methanol. Primary antibodies were then added for 45 min at 37°C. Unbound primary antibody was removed by three PBS washing steps. Binding of the primary antibody was detected with the appropriate FITC-conjugated secondary antibody (fluorescein isothiocyanate) (Dako, Germany) (45 min at 37°C). Counterstaining of cell nuclei was done with DAPI (4',6-diamidino-2-phenylindole). Images were collected using a Zeiss Axioplan 2 fluorescence microscope fitted with a Visitron Systems charge-coupled device camera (Puchheim, Germany). Images were processed using MetaView software and Adobe Photoshop.

### Plaque assay

MEF were seeded in 96-well plates (Ibidi, Germany) at a density of 4x10^4^ per well. 24 h later cells were infected with 2000 pfu/well of MCMV C3X-gfp using centrifugal enhancement (5min, 500xg) to enable synchronous infection. 4h later the inoculum was removed and cells were incubated in 300 μl/well medium containing 20 μg/ml mAb or serum at a dilution of 1:100. Infection was documented using a Leica DMI 6000B microscope starting at day 3 post infection. Magnification: 200fold. Filter: excitation 488 nm, emission 509, exposure times: 40 ms, picture size: 640 μm x 478 μm.

### Generation of the MCMV gB model

The model of the MCMV gB structure was generated by standard homology modelling procedures using the program MODELLER 9.10 [68] based on a sequence alignment with the template structure of HCMV gB (PDB code: 5CXF). Two loop regions (Asn409-Gln410 and Lys435-Val475 of HCMV gB) were not resolved in the reference structure and were therefore not modelled. Images were generated with VMD 1.9.1 [69].

### Statistical analysis

Statistical analysis was performed by one way ANOVA using Bonferroni´s multiple comparison test using GraphPad Prism (version 6; GraphPad Software, USA).

## Supporting information

S1 Fig Box plots of reduction in viral load compared to isotype control after administration of neutralizing mAbs. Viral load in animals treated with the isotype control was set to 100% and used to calculate the reduction in animals treated with mAbs or immune serum. Statistics: One way ANOVA using Bonferroni´s multiple comparison test *: p<0.05, **: p<0.01, ***: p<0.001, ****:p<0.0001.
(PDF)

S2 Fig Negative correlation of *in-vivo* protective capacity with *in-vivo* serum half-life of monoclonal antibodies. mAbs were injected i.p. into mice and serum was obtained at day 1 and at day 4 after injection. *In-vitro* virus neutralization activity was determined in the sera and the decay of 50% neutralization titer of the sera from day 1 to day 4 is displayed versus the reduction of viral load (as compared to control infected mice) in lung, liver, salivary gland, kidney and spleen when the mice were sacrificed on day 10 post infection. Each symbol corresponds to one organ (mean of eight mice). mAb designations are depicted above the cohorts of symbols. The decay of antibodies in the serum negatively correlated with the reduction of virus load (r^2^ = 0.48, p<0.001, linear regression).
(PDF)

S3 Fig Box plots of reduction in viral load compared to isotype control after administration of non-neutralizing mAbs. Viral load in animals treated with the isotype control was set to 100% and used to calculate the reduction in animals treated with mAbs or immune serum. Statistics: One way ANOVA using Bonferroni´s multiple comparison test *: p<0.05, **: p<0.01, ***:p<0.001, ****:p<0.0001.
(PDF)

S4 Fig Binding competition of gB-specific mAbs. Individual mAbs were biotinylated using a commercial kit (EZ Link® Sulfo-NHS Biotinylation Kit, Thermo Fisher Scientific) according to the suggestions of the manufacturer. Biotinylated mAbs were titrated to determine concentrations within the linear response range to the antigen. A fixed amount of a single biotinylated mAb (b) was then used in competition ELISA using increasing concentrations of competitor mAb as shown on the x-axis. Binding was detected using HRP-conjugated Streptavidin (Thermofisher, Germany).
(PDF)

S5 Fig Percent reduction in viral load following administration of mAbs compared to administration of immune serum. Reduction in viral load in animals treated with immune serum was set to 100% and used to calculate the reduction in animals treated with individual mAbs or the mAb combination M11 + 97.3 (nt combi).
(PDF)

S6 Fig Survival after prophylactic application of antibodies. 250 μg IgG per mouse was applied one day before infection with 104 pfu of MCMV157luc. Survival was monitored for 100 days p.i. Statistics: log-rank (Mantel-Cox) test: p <0.0001. Representative data from 2 independent experiments.
(PDF)

S7 Fig (A) Neutralization titer of mAbs 97.3, M11 and a mixture of both mAbs.
(B) Neutralization titer of mouse sera one day after after adoptive transfer of mAb combination 97.3 plus M11. Neutralization titer was determined in-vitro on murine fibroblasts using MCMV157luc.
Individual mice are indicated by number and color. Dotted line: 50% neutralization.
(PDF)
