# Supplementary material for: Protective capacity of neutralizing and non-neutralizing antibodies against glycoprotein B of cytomegalovirus
Source: PLoS Pathog. 2017 Aug 30;13(8):e1006601. doi: 10.1371/journal.ppat.1006601 (PMC5595347; doi:10.1371/journal.ppat.1006601)
Supplement: S2 Fig — mAbs were injected i.p. into mice and serum was obtained at day 1 and at day 4 after injection. In-vitro virus neutralization activity was determined in the sera and the decay of 50% neutralization titer of the sera from day 1 to day 4 is displayed versus the reduction of viral load (as compared to control infected mice) in lung, liver, salivary gland, kidney and spleen when the mice were sacrificed on day 10 post infection. Each symbol corresponds to one organ (mean of eight mice). mAb designations are depicted above the cohorts of symbols. The decay of antibodies in the serum negatively correlated with the reduction of virus load (r2 = 0.48, p<0.001, linear regression). (PDF) [file ppat.1006601.s002.pdf]

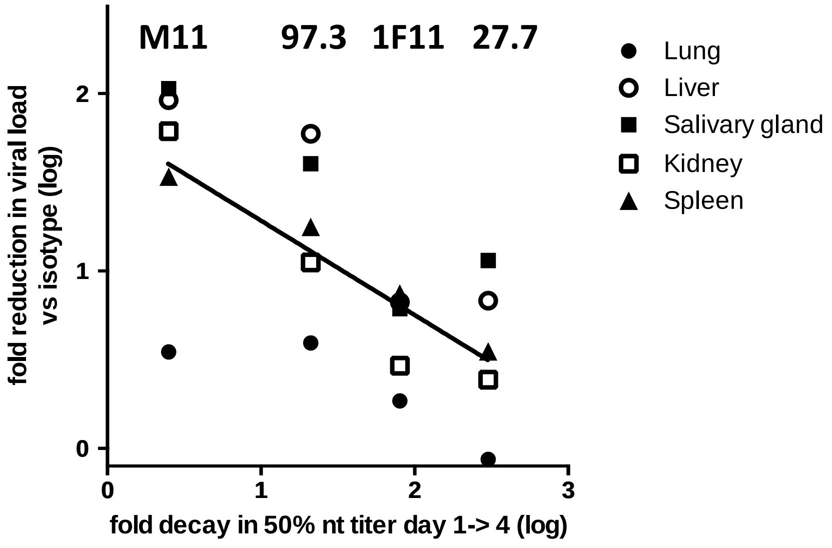

Supplemental figure S2

Negative correlation of *in-vivo* protective capacity with *in-vivo* serum half-life of monoclonal antibodies.

mAbs were injected i.p. into mice and serum was obtained at day 1 and at day 4 after injection. *In-vitro* virus neutralization activity was determined in the sera and the decay of 50% neutralization titer of the sera from day 1 to day 4 is displayed versus the reduction of viral load (as compared to control infected mice) in lung, liver, salivary gland, kidney and spleen when the mice were sacrificed on day 10 post infection. Each symbol corresponds to one organ (mean of eight mice). mAb designations are depicted above the cohorts of symbols. The decay of antibodies in the serum negatively correlated with the reduction of virus load ($r^2=0.48$, $p<0.001$, linear regression).
