# Supplementary material for: Protective capacity of neutralizing and non-neutralizing antibodies against glycoprotein B of cytomegalovirus
Source: PLoS Pathog. 2017 Aug 30;13(8):e1006601. doi: 10.1371/journal.ppat.1006601 (PMC5595347; doi:10.1371/journal.ppat.1006601)
Supplement: S3 Fig — Viral load in animals treated with the isotype control was set to 100% and used to calculate the reduction in animals treated with mAbs or immune serum. Statistics: One way ANOVA using Bonferroni´s multiple comparison test *: p<0.05, **: p<0.01, ***:p<0.001, ****:p<0.0001. (PDF) [file ppat.1006601.s003.pdf]

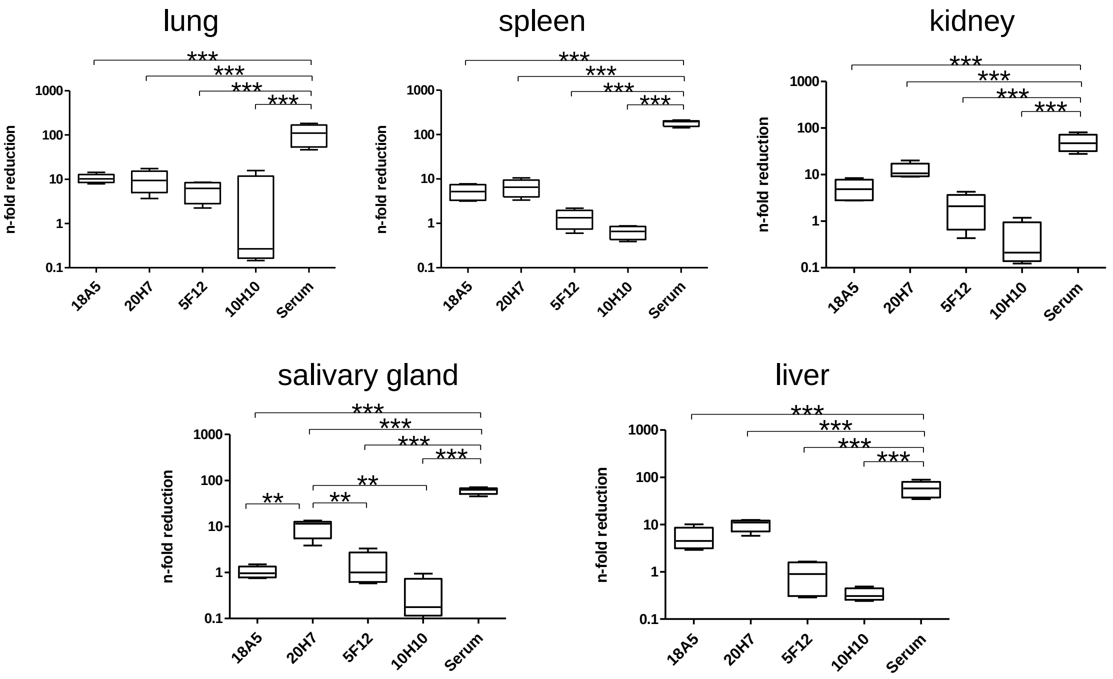

Supplemental figure S3
Box plots of reduction in viral load compared to isotype control after administration of nonneutralizing mAbs. Viral load in animals treated with the isotype control was set to 100% and used to calculate the reduction in animals treated with mAbs or immune serum. Statistics: One way ANOVA using Bonferroni´s multiple comparison test *: $p<0.05$, **: $p<0.01$, ***:$p<0.001$, ****:$p<0.0001$.
