# Supplementary material for: Protective capacity of neutralizing and non-neutralizing antibodies against glycoprotein B of cytomegalovirus
Source: PLoS Pathog. 2017 Aug 30;13(8):e1006601. doi: 10.1371/journal.ppat.1006601 (PMC5595347; doi:10.1371/journal.ppat.1006601)
Supplement: S4 Fig — Individual mAbs were biotinylated using a commercial kit (EZ Link® Sulfo-NHS Biotinylation Kit, Thermo Fisher Scientific) according to the suggestions of the manufacturer. Biotinylated mAbs were titrated to determine concentrations within the linear response range to the antigen. A fixed amount of a single biotinylated mAb (b) was then used in competition ELISA using increasing concentrations of competitor mAb as shown on the x-axis. Binding was detected using HRP-conjugated Streptavidin (Thermofisher, Germany). (PDF) [file ppat.1006601.s004.pdf]

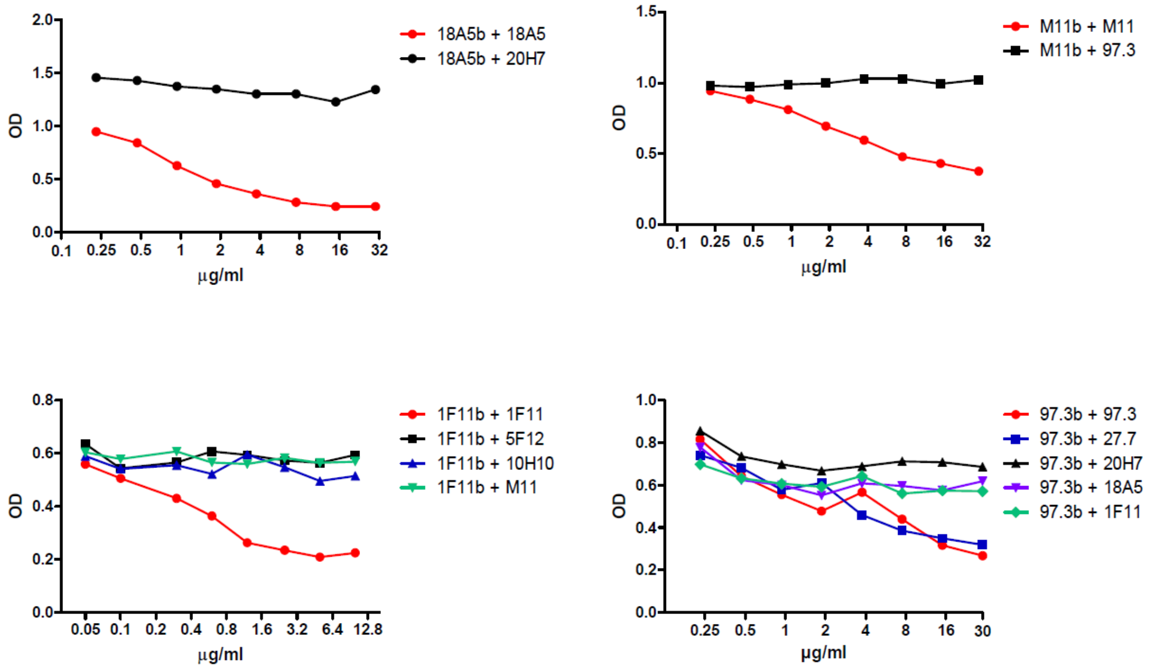

Supplemental figure S4

Binding competition of gB-specific mAbs.

Individual mAbs were biotinylated using a commercial kit (EZ Link® Sulfo-NHS Biotinylation Kit, Thermo Fisher Scientific) according to the suggestions of the manufacturer. Biotinylated mAbs were titrated to determine concentrations within the linear response range to the antigen. A fixed amount of a single biotinylated mAb (b) was then used in competition ELISA using increasing concentrations of competitor mAb as shown on the x-axis.
