# Supplementary material for: Protective capacity of neutralizing and non-neutralizing antibodies against glycoprotein B of cytomegalovirus
Source: PLoS Pathog. 2017 Aug 30;13(8):e1006601. doi: 10.1371/journal.ppat.1006601 (PMC5595347; doi:10.1371/journal.ppat.1006601)
Supplement: S5 Fig — Reduction in viral load in animals treated with immune serum was set to 100% and used to calculate the reduction in animals treated with individual mAbs or the mAb combination M11 + 97.3 (nt combi). (PDF) [file ppat.1006601.s005.pdf]

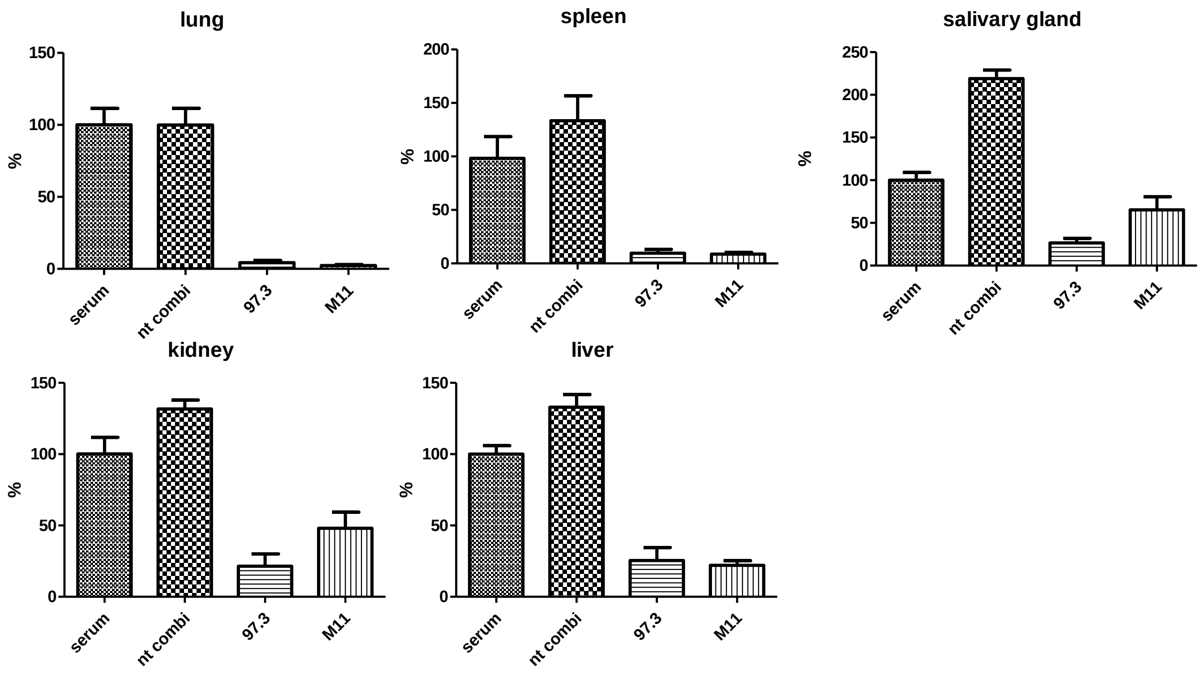

Supplemental figure S5

Relative reduction in viral load by mAbs compared to immune serum.
Reduction in viral load following treatment with serum was set to 100% and the reduction after treatment with the mAb combination 97.3+M11 (nt combi) or individual mAbs (97.3; M11) was calculated. Combined data from 2 experiments. Columns: Mean/SD
