# Supplementary material for: Protective capacity of neutralizing and non-neutralizing antibodies against glycoprotein B of cytomegalovirus
Source: PLoS Pathog. 2017 Aug 30;13(8):e1006601. doi: 10.1371/journal.ppat.1006601 (PMC5595347; doi:10.1371/journal.ppat.1006601)
Supplement: S6 Fig — 250 μg IgG per mouse was applied one day before infection with 104 pfu of MCMV157luc. Survival was monitored for 100 days p.i. Statistics: log-rank (Mantel-Cox) test: p <0.0001. Representative data from 2 independent experiments. (PDF) [file ppat.1006601.s006.pdf]

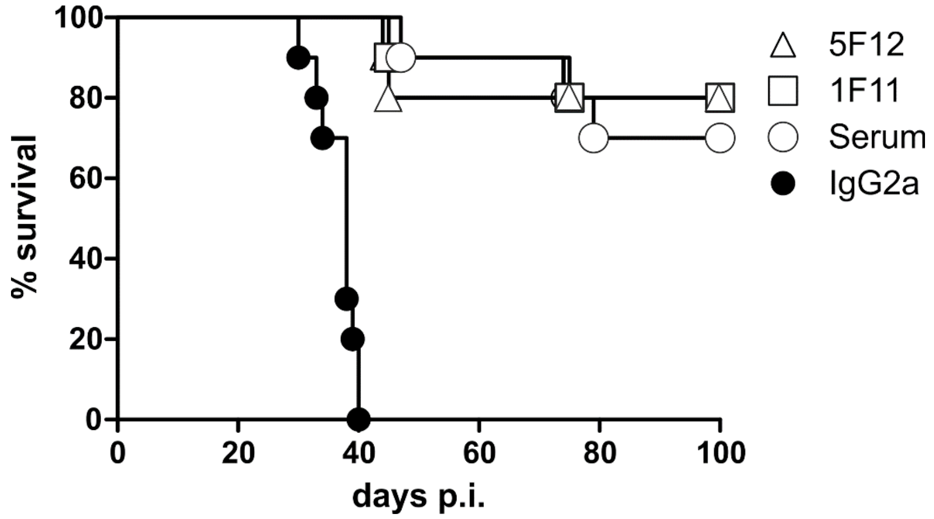

Supplemental figure S6

Survival after prophylactic application of antibodies.

250 µg IgG per mouse was applied one day before infection with 10^4 pfu of MCMV157luc. Survival was monitored for 100 days p.i. Statistics: log-rank (Mantel-Cox) test: $p < 0.0001$. Representative data from 2 independent experiments.
