# Supplementary material for: Protective capacity of neutralizing and non-neutralizing antibodies against glycoprotein B of cytomegalovirus
Source: PLoS Pathog. 2017 Aug 30;13(8):e1006601. doi: 10.1371/journal.ppat.1006601 (PMC5595347; doi:10.1371/journal.ppat.1006601)
Supplement: S7 Fig — (A) Neutralization titer of mAbs 97.3, M11 and a mixture of both mAbs. (B) Neutralization titer of mouse sera one day after after adoptive transfer of mAb combination 97.3 plus M11. Neutralization titer was determined in-vitro on murine fibroblasts using MCMV157luc. Individual mice are indicated by number and color. Dotted line: 50% neutralization. (PDF) [file ppat.1006601.s007.pdf]

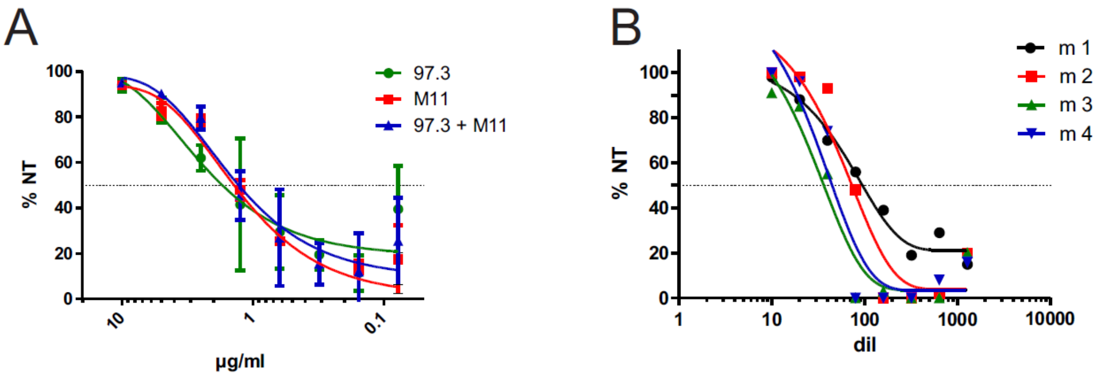

Supplemental figure S7

(A) Neutralization titer of mAbs 97.3, M11 and a mixture of both mAbs.

(B) Neutralization titer of mouse sera one day after after adoptive transfer of mAb combination 97.3 plus M11. Neutralization titer was determined *in vitro* on murine fibroblasts using MCMV157luc (). Individual mice are indicated by number and color. Dotted line: 50% neutralization.
